# A Novel, Integrative Approach for Evaluating Progression in Multiple Sclerosis: Development of a Scoring Algorithm

**DOI:** 10.2196/17592

**Published:** 2020-04-14

**Authors:** Chloe Tolley, Daniela Piani-Meier, Sarah Bentley, Bryan Bennett, Eddie Jones, James Pike, Frank Dahlke, Davorka Tomic, Tjalf Ziemssen

**Affiliations:** 1 Adelphi Values Ltd Macclesfield United Kingdom; 2 Novartis Pharma AG Basel Switzerland; 3 Adelphi Real World Ltd Macclesfield United Kingdom; 4 Center of Clinical Neuroscience Neurological University Clinic Carl Gustav Carus TU Dresden Dresden Germany

**Keywords:** multiple sclerosis, relapsing-remitting, SPMS, tool, algorithm, disease progression

## Abstract

**Background:**

There is an unmet need for a tool that helps to evaluate patients who are at risk of progressing from relapsing-remitting multiple sclerosis to secondary progressive multiple sclerosis (SPMS). A new tool supporting the evaluation of early signs suggestive of progression in multiple sclerosis (MS) has been developed. In the initial stage, concepts relevant to progression were identified using a mixed method approach involving regression on data from a real-world observational study and qualitative research with patients and physicians. The tool was drafted in a questionnaire format to assess these variables.

**Objective:**

This study aimed to develop the scoring algorithm for the tool, using both quantitative and qualitative research methods.

**Methods:**

The draft scoring algorithm was developed using two approaches: quantitative analysis of real-world data and qualitative analysis based on physician interviews and ranking and weighting exercises. Variables that were included in the draft tool and regarded as most clinically relevant were selected for inclusion in a multiple logistic regression. The analyses were run using physician-reported data and patient-reported data. Subsequently, a ranking and weighting exercise was conducted with 8 experienced neurologists as part of semistructured interviews. Physicians were presented with the variables included in the draft tool and were asked to rank them in order of strength of contribution to progression and assign a weight by providing a percentage of the overall contribution. Physicians were also asked to explain their ranking and weighting choices. Concordance between physicians was explored.

**Results:**

Multiple logistic regression identified age, MS disease activity, and Expanded Disability Status Scale score as the most significant physician-reported predictors of progression to SPMS. Patient age, mobility, and self-care were identified as the strongest patient-reported predictors of progression to SPMS. In physician interviews, the variables ranked and weighted as most important were stability or worsening of symptoms, intermittent or persistent symptoms, and presence of ambulatory and cognitive symptoms. Across all physicians, the level of concordance was 0.278 (*P*<.001), indicating a low to moderate, but statistically significant, level of agreement. Variables were categorized as high (n=8), moderate (n=8), or low (n=10) importance based on the findings from the different approaches described above. Accordingly, the respective questions in the tool were assigned a weight of “three,” “two,” or “one” to inform the draft scoring algorithm.

**Conclusions:**

This study further confirms the need for a tool to provide a consistent, comprehensive approach across physicians to support the early evaluation of signs indicative of progression to SPMS. The novel and comprehensive approach to develop the draft scoring algorithm triangulates data obtained from ranking and weighting exercises, qualitative interviews, and a real-world observational study. Variables that go beyond the clinically most obvious impairment in lower limbs have been identified as relevant subtle/sensitive signs suggestive of progressive disease.

## Introduction

### Background

Onset of secondary progressive disease course is associated with an unfavorable and severe long-term outcome in multiple sclerosis (MS) [1], and there are no distinct biomarkers or clinical criteria to detect the transition to secondary progressive multiple sclerosis (SPMS). Diagnosis of SPMS is usually retrospective in nature and based on the identification of progression independent of relapses [2], often relying on patients’ recollection of worsening of their clinical status as well as the thoroughness of physicians’ inquiries at the regular visits [3]. There is a period of diagnostic uncertainty, which lasts for an average of 3 years [4]. Lack of treatment options, psychological burden imposed on the patients, and concerns regarding reimbursement are additional challenges toward making a definitive diagnosis [5,6].

With the advent of newer and highly effective therapies, recognizing early indicators of progressive disease may represent a window of opportunity for intervention [4]. A tool that helps to assess the signs of progression may support an early identification of patients who are at a higher risk of transitioning to SPMS. In the past, several studies have evaluated various clinical and magnetic resonance imaging (MRI) variables predictive of the risk of secondary progression based exclusively on empirical or quantitative assessments of different study cohorts [1,7-13]. Some of those studies further developed models or algorithms, predicting the risk of conversion to SPMS—Skoog et al (MS prediction) [12], Manouchehrinia et al (SPMS nomogram) [11], and Lorscheider et al (calculators) [10]. The parameters identified as relevant for conversion are not consistent across the different studies probably because of the differences in their respective study settings, used datasets, and methodologies.

### Objective

We conducted a comprehensive research study using a mixed methods approach for developing a new tool to support the early evaluation of signs of progressive disease. As a first step, the tool content was developed in the form of a questionnaire based on the results obtained from regression analysis on data from a real-world observational study and insights obtained from the open-ended, qualitative, concept elicitation interviews with patients and physicians [14]. Here, we describe the next stage of the research, which aimed to develop the scoring algorithm for the tool by determining the relevance and importance of each item included in the questionnaire, using a mixed methods approach.

## Methods

### Scoring Algorithm Development

**Figure 1:**
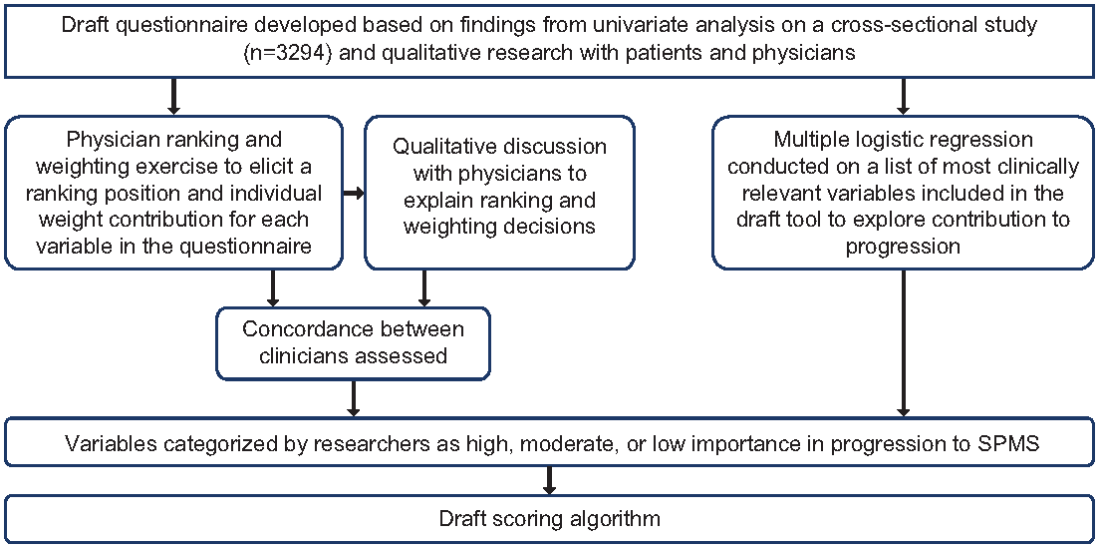
Overview of the development of the scoring algorithm. SPMS: secondary progressive multiple sclerosis.

The draft scoring algorithm was developed using two approaches: quantitative analysis of real-world data and qualitative analysis based on physician interviews and ranking and weighting exercises (Figure 1). Quantitative methods involved retrospective analysis on data from a global cross-sectional study that collected information from physicians (neurologists) and their consulting MS patients on demographics, clinical history, current symptomatology, treatment history, and quality of life [14]. The study was run without set hypothesis before data collection but involved a large number of MS patients (n=3294) in a real-world setting, across countries, reflecting clinical practice and physician views. In the previous study, univariate analysis was conducted on variables included in the observational study [14]. Multivariate regression analysis was used in this study to determine variables associated with being early relapsing-remitting multiple sclerosis (RRMS) or early SPMS. In an iterative approach, these findings were used alongside qualitative research to inform the development of the draft tool content. The development and content of the draft tool (in the form of a questionnaire) have been described in detail previously by Ziemssen et al [14].

### Assigning Rank and Weights

Physician ranking and weighting exercises were conducted as part of a qualitative interview. Eight physicians in Germany (n=4) and the United States (n=4), all neurologists, were recruited into this study by specialist recruitment agencies. Physicians were required to meet prespecified eligibility criteria (Multimedia Appendix 1). Each physician participated in a 45-min, face-to-face, semistructured, qualitative interview, conducted by a trained interviewer. First, physicians were presented with the list of variables included in the tool (Multimedia Appendix 2) and were asked to rank them in order of how strongly they contribute to SPMS progression. Then, physicians were asked to provide a “weight” for each variable by dividing 100 plastic tokens among the variables to indicate the contribution each variable should have to make up the total percentage score. Throughout the tasks, physicians were encouraged to “think aloud” and provide a rationale for the decisions that they made. Following completion of each task, physicians were asked to further explain their rankings or weightings or to clarify any decisions that they had not already commented on. In addition, physicians were asked to comment on the ease of completion of the tasks and to report if any important variables were missing. Mean and range weighting and ranking positions were produced for each variable.

All interviews were audio-recorded and transcribed verbatim. Physicians’ rationales for ranking and weighting choices were analyzed using thematic analysis on Atlas.ti software [15]. Furthermore, the level of agreement between physicians for the ranking of variables was investigated at the individual country level (Germany and the United States) and for all physicians combined. Kendall coefficient of concordance was used to assess the agreement between the ranked concepts (from most important to least important). The test statistic, Kendall W, is calculated between 0 and 1, where 0 indicates no agreement between raters and 1 indicates complete agreement.

Variables were categorized by researchers as high, moderate, or low in importance, based on the review of the findings from quantitative regression analysis, the ranking and weighting exercise, and the qualitative physicians’ rationale for the ranks and weights. A scoring algorithm was then developed to produce a total score for the draft tool.

## Results

### Regression Analysis

**Figure 2:**
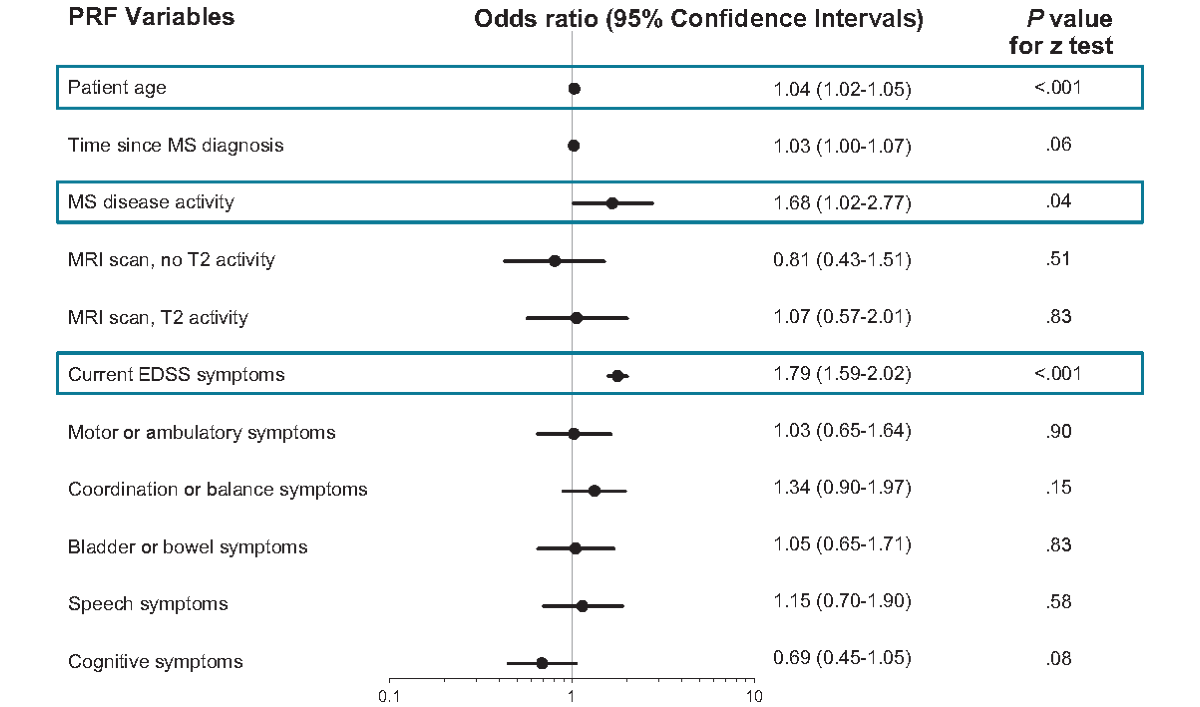
Multivariate regression analysis: variables that are predictors of progression to secondary progressive multiple sclerosis. Disease activity is Physician-reported multiple sclerosis disease activity based on the physician’s overall perception of the patient’s disease activity, ranging from “no activity to high activity” (no specific definition of disease activity was provided to the physicians); an odds ratio >1 implies a higher risk of secondary progressive multiple sclerosis; the blue box highlights the significant predictors. EDSS: Expanded Disability Status Scale; MRI: magnetic resonance imaging; MS: multiple sclerosis; PRF: patient record form; SPMS: secondary progressive multiple sclerosis; T2: transverse relaxation time. Black dots indicate odds ratio (point estimate); black line indicates the 95% confidence interval.

**Figure 3:**
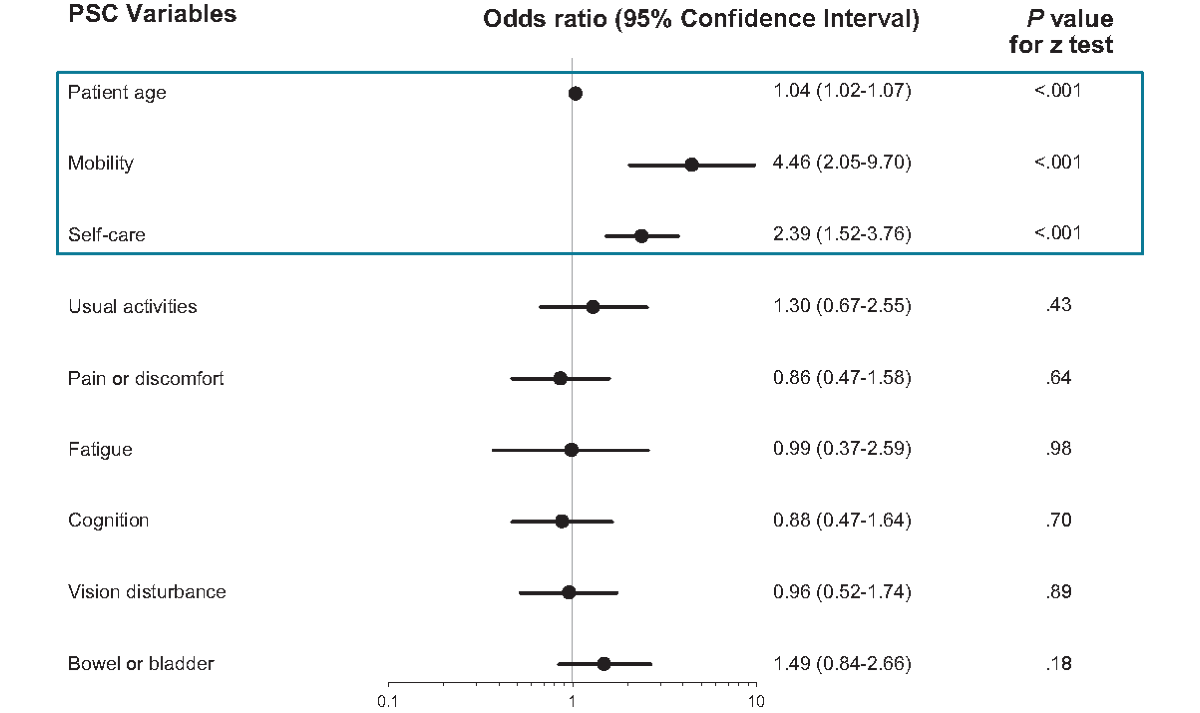
Multivariate regression analysis: patient self-completion form variable. An odds ratio >1 implies a higher risk of secondary progressive multiple sclerosis; the blue box highlights the significant predictors. PSC: patient self-completion. Black dots indicate odds ratio (point estimate); black line indicates the 95% confidence interval.

A total of 11 physician-reported variables and nine patient-reported variables were identified for inclusion in multiple logistic regression analyses. Age (odds ratio [OR] 1.04; *P*<.001), MS disease activity (OR 1.68; *P*<.05), and Expanded Disability Status Scale (EDSS) score (OR 1.79; *P*<.001) were identified as the most significant physician-reported predictors of progression to SPMS (Figure 2). Patient age (OR 1.05; *P*<.001), mobility (OR 4.46; *P*<.001), and self-care (OR 2.39; *P*<.001) were identified as the strongest patient-reported predictors of progression to SPMS (Figure 3).

### Qualitative Interviews

#### Demographics

Physicians had a range of demographic characteristics and clinical experience. The sample consisted of 5 male and 3 female physicians, all of whom were neurologists. The mean age of the sample was 50.2 years (range 38-69). US physicians had been in their role for an average of 20 years (range 7-41), whereas German physicians had been in their role for an average of 8 years (range 3-21). The physicians were employed in a range of settings, including private practice (5/8), hospital-based care (2/8), and academia (2/8). On average, physicians saw 18 RRMS patients and 3 SPMS patients per week; German physicians saw more RRMS and SPMS patients per week than the US physicians. On average, physicians estimated that 30.6% (range 7%-70%) of their workload was dedicated to patients with MS.

#### Ranking and Weighting

The average ranking and weighting was calculated for each variable, and the top 10 ranked and weighted variables were identified. Findings from physician-completed ranking and weighting exercises were consistent in that 7 of the top 10 variables were present in both the ranked and weighted list. The top 10 variables included improvement, stability, or worsening of symptoms; intermittence or persistence of symptoms; ambulatory symptoms; cognitive symptoms; EDSS score; mobility; and presence or absence of relapse (Table 1). Variables that were in the top 10 for both the ranking and weighting exercises are italicized. Lower ranking indicates greater importance. Higher weighting indicates greater importance.

**Table 1:** Top 10 ranked and weighted variables.

Variable	Average rank	Variable	Average weight
*Improving, stable, or worsening* ^a^	5.1	*Improving, stable, or worsening*	9.9
*Intermittent or persistent*	6.9	*Intermittent or persistent*	6.4
*Ambulatory symptoms*	8.3	New magnetic resonance imaging activity	6.2
*Cognitive symptoms*	8.9	*Cognitive symptoms*	5.9
*EDSS* ^b^ *score*	10.1	*Mobility*	5.5
Time since diagnosis	10.4	*Ambulatory symptoms*	5.2
*Mobility*	10.6	*EDSS score*	5.2
Number of relapses	10.8	*Any relapses*	5.1
Motor symptoms	11.1	Coordination symptoms	4.8
*Any relapses*	11.2	Daily activities	4.7

^a^Italicized variables were among the top 10 variables in both the ranking and weighting exercise.

^b^EDSS: Expanded Disability Status Scale.

#### Categorizing Variables

**Figure 4:**
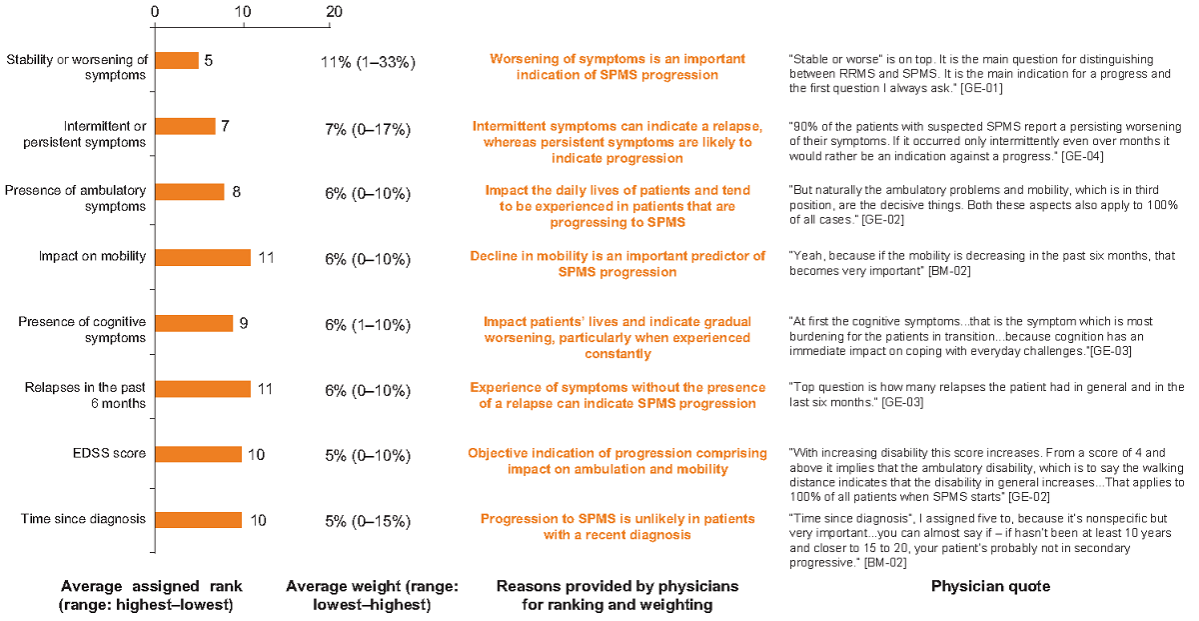
Variables of high importance in progression to secondary progressive multiple sclerosis. Ranking out of 26 variables included. Lower ranking indicates greater importance. EDSS: Expanded Disability Status Scale; RRMS: relapsing-remitting multiple sclerosis; SPMS: secondary progressive multiple sclerosis.

On the basis of the review of the findings from quantitative regression analysis, the ranking and weighting exercise, and the qualitative physicians’ rationale for rankings and weightings, eight variables were categorized by researchers as highly important in identifying progression to SPMS. These included variables describing the nature of the symptoms (intermittent vs persistent, stable vs worsening, and the absence or presence of relapses) and the presence of ambulatory, mobility, and cognitive symptoms, in addition to the EDSS score and time since diagnosis. Physicians explained that the variables rated as high importance were often indicators of progression to SPMS (Figure 4).

Eight variables were categorized as moderately important indicators of progression to SPMS, as determined by the qualitative findings and physician’ rankings and weightings. Moderately important variables included those relating to the characteristics of relapse (recovery from the most recent relapse, number of relapses in the past 6 months, and symptoms during relapse), the presence of specific symptoms (motor, coordination and balance, and speech), an objective clinical measure of progression (signs of new activity based on MRI scans), and the impact on daily activities.

**Figure 5:**
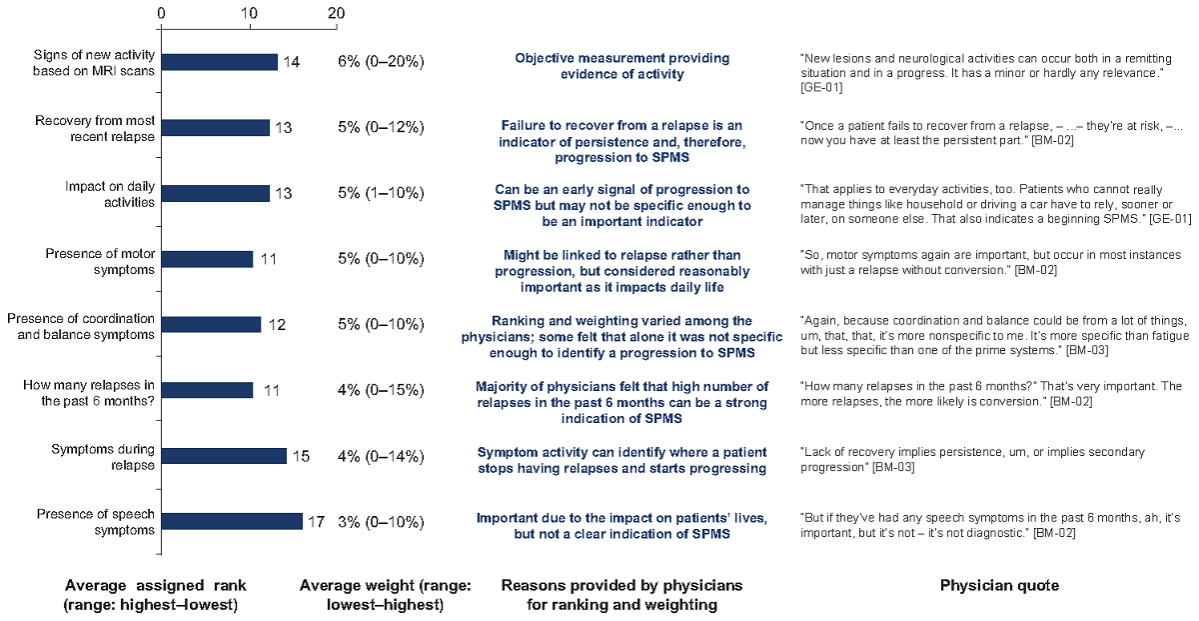
Variables of moderate importance in progression to secondary progressive multiple sclerosis. Ranking out of 26 variables included. Lower ranking indicates greater importance. MRI: magnetic resonance imaging; SPMS: secondary progressive multiple sclerosis.

Physicians explained that variables of moderate importance could be early signs of progression to SPMS but were not specific enough to be considered as highly important indicators (Figure 5).

**Figure 6:**
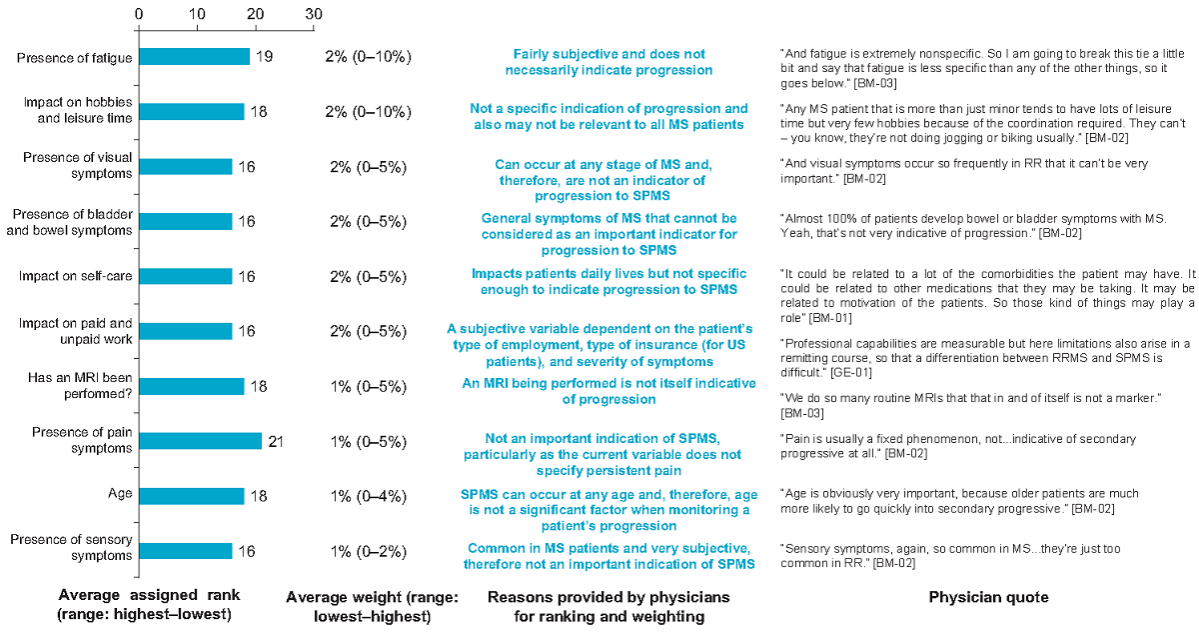
Variables of low importance in progression to secondary progressive multiple sclerosis. Ranking out of 26 variables included. Lower ranking indicates greater importance. SPMS: secondary progressive multiple sclerosis.

A total of 10 variables were categorized to be low indicators of progression to SPMS, as determined by physician rankings and weightings. These included fatigue, visual symptoms, bladder and bowel symptoms, pain, specific impacts (hobbies and leisure time, self-care, and work), and whether an MRI had been performed. Physicians explained that variables of low importance were subjective, general symptoms of MS, not relevant enough to MS and too unspecific for the progression to SPMS (Figure 6).

The majority of physicians reported that they found the task challenging, given the complex nature of identifying progression to SPMS. One physician suggested including medication history, and another physician suggested removing ambulatory symptoms as it is similar to impact on mobility.

Across all 8 physicians, the level of concordance was 0.278 (*P<*.001), indicating a low to moderate, but statistically significant, level of agreement. Physicians demonstrated slightly greater concordance within countries (United States: 0.42, *P=*.02; Germany: 0.385, *P=*.04; Table 2).

**Table 2:** Regional concordance across physicians.

Country	Rank analysis	
	Kendall W	*P* value
United States	0.42	.02
Germany	0.385	.04
All	0.278	*<*.001

#### Scoring Algorithm

On the basis of the results from the review of previous findings outlined by Ziemssen et al [14], regression analysis of observational study data, physician ranking and weighting, and the associated rationales, questions were weighted as follows:

- 3 for variables that were found to be important
- 2 for variables that were found to be moderately important
- 1 for variables that were found to be less important.

These weightings were integrated accordingly in the scoring algorithm. Absence of relapse; presence of motor, ambulatory, and cognitive symptoms; and the persistent worsening of any symptom were assigned the highest weights in the scoring algorithm.

Question scores were multiplied by question weight to produce a total score for that question. The weightings and maximum score for each section are shown in Multimedia Appendix 3. The standardized total score was calculated by summing the score for each section and reweighting to a score divided by 100.

## Discussion

### Overview

Disability progression is known to be a continuous process that starts very early in the disease course, identifiable even at Disability Status Scale score of 2 [16]. This is also evident from the similar rates of brain atrophy seen at the earliest vs later stages of MS [17]. In addition, cognitive impairment is also seen early in the disease course, including patients with clinically isolated syndrome [18]. Therefore, it is important to identify the signs of progression early, as the timing will determine the extent of therapeutic benefits and affect long-term outcome [19]. Currently, no established tools are available for use in routine clinical practice to support real-time “systematic” comprehensive assessment to help assess the subtle signs of progression [20]. During the stage 1 of our study, physicians confirmed the unmet need for such a tool in routine clinical practice and highlighted that a digital tool generating a score or a graphical output would be preferred and useful for clinical practice [14].

### Principal Findings

The quantitative and qualitative approaches employed in this study informed categorization of the variables in the draft questionnaire as of “high,” “moderate,” and “low” importance. As expected, differences between the categories were not pronounced, but it is noteworthy that the variables rated as highly important were consistent and presented substantial overlap, thus providing confidence in the categorization. In line with previous studies, ambulation, mobility, and EDSS score were identified, not unsurprisingly, as the most “obvious” parameters associated with progression across all approaches. Interestingly, cognition emerged as an additional highly relevant symptom associated with progression. This is consistent with the previous reports showing that cognition is impaired very early in the disease course, even before physical disability might be obvious. The cognitive impairment affects multiple functionalities and can negatively impact patients’ lives. In addition, cognition has been reported to be predictive of disease evolution [19,21], whereas cognitive reserve can be a buffer to disease progression, reflecting the ability to compensate for progressive injury and as a marker of neuronal network efficiency [22-24].

A 10-year follow-up study in patients with RRMS reports that patients with cognitive impairment are at a higher risk of reaching important milestone EDSS compared with cognitively preserved patients, and better cognitive performance at baseline was significantly predictive of lower SPMS conversion rates [25]. However, the ranking and weighting of variables by experienced neurologists clearly identified and confirmed the nature of the symptoms (eg, persistent worsening of any symptom) as the most important indicator of progression in MS, even more than a specific symptom itself, similar to the previous qualitative assessments with both physicians and patients [14].

Existing research into predictors of SPMS has been primarily quantitative, based on single-center or large-scale observational cohort studies [11,12]. Hence, variables identified as significant predictors are those typically based on objective, clinical observations collected as part of those specific electronic medical records applied and accessible in those registries [10-12]. Although the global cross-sectional study described in this paper involved a large number of MS patients in a real-world setting and reflected clinical practice and physician views, specific limitations were identified. Namely, more frequently consulting patients were more likely to participate, physicians were included only if they saw a minimum number of patients and were willing to take part, data accuracy relied on the reporting accuracy of the physician, and analyses were limited to the variables and information collected in the cross-sectional study. Furthermore, regression analysis, when using cross-sectional data, cannot prove a causal relationship but will be able to show an association between the outcome and study group that is independent of confounding factors.

Our study overcomes some of the limitations identified from these earlier studies, in that a more comprehensive approach was taken to identify the variables, also considering the descriptive and qualitative patient data assessed in daily practice and further ascertaining the importance of a particular variable for progression using a mixed methods approach. This enabled each variable to be classified by the level of contribution to progression thereby characterizing a sensitive algorithm that provides a score indicating the likelihood of progression for easy adoption in routine clinical practice. More importantly, none of the earlier studies evaluate progression at the current moment with such accuracy; rather, they provide a risk or likelihood of progression in the next few years or in the future.

Findings from the previous qualitative interviews with physicians showed a lack of consistency in the diagnosis and time taken to determine SPMS. The level of concordance in ranking and weighting among physicians in this study was low to moderate but statistically significant and with greater level concordance among physicians within countries (United States or Germany). The variation seen in this study confirms the lack of clear consensus and, hence, the unmet need for a universal standardized method, or tool, that supports the identification of patients at risk of progression. Despite this variation, the fact that there was a significant agreement between physicians on the importance of variables supports feasibility and the value of the data in developing an algorithm for the tool by identifying prevailing common concepts driving the physician to determine that the patient has progressed to SPMS.

As we used a mixed methods approach, some of the variables included in the tool were not collected in the RWE study and, thus, were categorized solely based on the ranking and weighting exercise and qualitative insights complimenting the findings from the regression analysis. The sample size for the qualitative assessment might have affected the level of agreement, and eventually, a more accurate representation of the level of agreement may have been achieved with a larger sample as any outliers in this sample had a large impact on the overall concordance statistic. However, as between and within differences in determining SPMS were also identified in earlier work and the MS neurologists in this study were all well experienced, it is unlikely that the level of agreement would have been a lot stronger with a larger sample size. By the inclusion of different geographies, we tried to cover for some of the differences in the prevailing health care systems and approaches adopted for the overall management of the disease.

Subsequent work confirmed the validity of the scoring algorithm derived from these analyses in a real-world setting and determined cutoffs to accurately differentiate between RRMS and SPMS patients with high specificity and sensitivity, in addition to evaluation of other measurement properties including interrater reliability [26]. The final validated MS Progression Discussion tool can be accessed on the Web [27].

### Conclusions

This study confirms the need for a tool to support the early evaluation of signs of progression to SPMS. The novel and comprehensive approach to develop the draft scoring algorithm triangulates data obtained from ranking and weighting exercises, qualitative interviews, and a real-world observational study. Variables that go beyond the clinically most obvious impairment in lower limbs have been identified as relevant subtle or sensitive signs suggestive of progressive disease. and have been integrated in the algorithm. The tool might, therefore, contribute to a more comprehensive physician-patient interaction in evaluating a patient’s current disease status and level of progression. Future work will aim to validate this scoring algorithm longitudinally in a real-world setting and its suitability for longitudinal monitoring of disease symptoms and its impacts.

## Appendix

Multimedia Appendix 1 Physician eligibility criteria.

Multimedia Appendix 2 Variables rated by physicians.

Multimedia Appendix 3 Scoring algorithm—question weights and total scores.

## Abbreviations

EDSS: Expanded Disability Status Scale
MRI: magnetic resonance imaging
MS: multiple sclerosis
OR: odds ratio
RRMS: relapsing-remitting multiple sclerosis
RWE: real-world evidence
SPMS: secondary progressive multiple sclerosis
