# Supplementary material for: A Novel, Integrative Approach for Evaluating Progression in Multiple Sclerosis: Development of a Scoring Algorithm
Source: JMIR Med Inform. 2020 Apr 14;8(4):e17592. doi: 10.2196/17592 (PMC7189255; doi:10.2196/17592)
Supplement: Multimedia Appendix 1 [file medinform_v8i4e17592_app1.docx]

## Multimedia Appendix 1: Physician eligibility criteria

Physicians must meet all of the following inclusion criteria to be eligible for enrolment into the study:

1. Physician is a specialist neurologist
2. Physician is currently responsible for the care and management of patients with MS
3. Physician sees >10 patients with MS per week
4. Physician is willing and able to provide written informed consent and attend and participate in a 45-minute face-to-face interview
5. Physician is literate in US English and verbally fluent in US English OR physician is literate in German and verbally fluent in German
