# Supplementary material for: A Novel, Integrative Approach for Evaluating Progression in Multiple Sclerosis: Development of a Scoring Algorithm
Source: JMIR Med Inform. 2020 Apr 14;8(4):e17592. doi: 10.2196/17592 (PMC7189255; doi:10.2196/17592)
Supplement: Multimedia Appendix 2 [file medinform_v8i4e17592_app2.docx]

Multimedia appendix 2: Variables rated by physicians

| Relapses in the past 6 months |
| --- |
| Recovery from most recent relapse |
| Signs of new activity based on MRI scan (e.g. new or enlarging gadolinium-enhanced T1 weighted lesions or increased brain volume) |
| Presence of visual symptoms |
| Presence of motor symptoms |
| Presence of ambulatory symptoms |
| Presence of coordination and balance symptoms |
| Presence of pain symptoms |
| Presence of sensory symptoms |
| Presence of bladder and bowel symptoms |
| Presence of speech symptoms |
| Presence of cognitive symptoms |
| Presence of fatigue |
| Symptoms during relapse |
| Intermittent or persistent symptoms |
| Stability of symptoms |
| Impact on mobility |
| Impact on self-care |
| Impact on daily activities |
| Impact on hobbies and leisure time |
| Impact on paid and unpaid work |
| Age |
| EDSS score |
| Time since diagnosis |
