# Supplementary material for: A Novel, Integrative Approach for Evaluating Progression in Multiple Sclerosis: Development of a Scoring Algorithm
Source: JMIR Med Inform. 2020 Apr 14;8(4):e17592. doi: 10.2196/17592 (PMC7189255; doi:10.2196/17592)
Supplement: Multimedia Appendix 3 [file medinform_v8i4e17592_app3.docx]

## Multimedia appendix 3: Scoring algorithm

Scoring algorithm - question weights and total scores

| Question | | Response and score | Question weight | Maximum score |
| --- | --- | --- | --- | --- |
| **Disease activity** | | | |  |
| Relapse | | Yes=0  No=2 | 3 | 6 |
| Number of relapses | | 1=2  2=1 3 or more=0 | 2 | 4 |
| Degree of recovery | | Full recovery=0 Nearly full recovery=0 Partial recovery=1 A little recovery=1 No recovery = 2 | 2 | 4 |
| **Symptoms in the past 6 months** | | | |  |
| Visual Coordination/balance Pain Sensory Bladder and bowel Speech Fatigue | In the presence of relapse? | Yes=0 No=1 N/A=1* | 1 | 1 |
|  |  | Intermittent=0 Persistent=3 |  | 3 |
|  |  | Improving=0 Stable=0 Worsening=3 N/A=0 |  | 3 |
| Motor Ambulatory  Cognitive | In the presence of relapse? | Yes=0 No=1 N/A=1* | 2 | 2 |
|  |  | Intermittent=0 Persistent=3 |  | 6 |
|  | If persistent… | Improving=0 Stable=0 Worsening=3 N/A=0 |  | 6 |
| **Impacts in the past 6 months** | | | |  |
| Impact on mobility | | None=0  Little=1  Moderate=1  Severe=2  Unable to do this activity=2 | 2 | 4 |
| Impact on self-care | |  | 1 | 2 |
| Impact on daily activities | |  | 2 | 4 |
| Impact on hobbies and leisure | |  | 1 | 2 |
| Impact on paid and unpaid work | | None=0  Little=1  Moderate=1  Severe=2  Unable to do this activity=2  N/A = 0 | 1 | 2 |
| **Clinical variables** | | | | |
| EDSS | | EDSS score ≤3.5=1  EDSS score ≥4 and <6=2  EDSS score ≥6=3 | 1 | 3 |
| Age | | Age <45=0  Age ≥45=2 | 1 | 2 |
| Maximum score = question score multiplied by the question weight  * N/A response is selected automatically when the clinician has responded ‘no’ to the first disease activity question (relapse) | | | | |
